# Nerve Suture Combined With ADSCs Injection Under Real-Time and Dynamic NIR-II Fluorescence Imaging in Peripheral Nerve Regeneration *in vivo*

**DOI:** 10.3389/fchem.2021.676928

**Published:** 2021-07-14

**Authors:** Shixian Dong, Sijia Feng, Yuzhou Chen, Mo Chen, Yimeng Yang, Jian Zhang, Huizhu Li, Xiaotong Li, Liang Ji, Xing Yang, Yuefeng Hao, Jun Chen, Yan Wo

**Affiliations:** ^1^Department of Anatomy and Physiology, School of Medicine, Shanghai Jiao Tong University, Shanghai, China; ^2^Department of Sports Medicine, Sports Medicine Institute of Fudan University, Huashan Hospital, Fudan University, Shanghai, China; ^3^Department of Orthopedics, Affiliated Suzhou Hospital of Nanjing Medical University, Suzhou, China

**Keywords:** peripheral nerve injury, nerve suture, ADSCs, NIR-II, fluorescence imaging, nerve regeneration

## Abstract

Peripheral nerve injury gives rise to devastating conditions including neural dysfunction, unbearable pain and even paralysis. The therapeutic effect of current treatment for peripheral nerve injury is unsatisfactory, resulting in slow nerve regeneration and incomplete recovery of neural function. In this study, nerve suture combined with ADSCs injection was adopted in rat model of sciatic nerve injury. Under real-time visualization of the injected cells with the guidance of NIR-II fluorescence imaging *in vivo*, a spatio-temporal map displaying cell migration from the proximal injection site (0 day post-injection) of the nerve to the sutured site (7 days post-injection), and then to the distal section (14 days post-injection) was demonstrated. Furthermore, the results of electromyography and mechanical pain threshold indicated nerve regeneration and functional recovery after the combined therapy. Therefore, in the current study, the observed ADSCs migration *in vivo*, electrophysiological examination results and pathological changes all provided robust evidence for the efficacy of the applied treatment. Our approach of nerve suture combined with ADSCs injection in treating peripheral nerve injury under real-time NIR-II imaging monitoring *in vivo* added novel insights into the treatment for peripheral nerve injury, thus further enhancing in-depth understanding of peripheral nerve regeneration and the mechanism behind.

## Introduction

The incidence of peripheral nerve injury was ~13 to 23 per 100,000 persons per year (Evans, 2001; Taylor et al., 2008; Asplund et al., 2009), the consequences of which might cause lifelong disorder and permanent disability. Malfunction of peripheral nerves due to injury can lead to physiological disorders including paralysis, limb numbness, pain, etc. Various therapeutics have been employed for peripheral nerve regeneration for hundreds of years (Artico et al., 1996; Lundborg, 2000; Battiston et al., 2009; Pabari et al., 2010; Tang et al., 2013). Among them, surgical options serve to realign damaged nerves, through approximation of the nerve stumps by nerve suture (Sulaiman and Gordon, 2000; Evans, 2001; Campbell, 2008). However, the efficacy of pure nerve suture is unfortunately hindered, failing to achieve sufficient tissue regeneration and full neurofunctional recovery of the injured nerve (Lin et al., 2013; Carriel et al., 2014; Gu et al., 2014).

In recent years, adipose stem cells (ADSCs) have been extensively studied in the field of peripheral nerve regeneration (Zheng and Liu, 2019; Jahromi et al., 2020; Yamamoto et al., 2020; Zhou et al., 2020). In the perspective of acquisition, it is reported that 1 g of adipose tissue contains ~(0.5–2) × 10^6^ adipose cells, among which 1–10% are stem cells (Oedayrajsingh-Varma et al., 2006; Zhu et al., 2008). In comparison, roughly 6 × 10^6^ nucleated cells can be obtained from 1 ml of bone marrow, containing only 0.001–0.01% of stem cells (Pittenger et al., 1999; Salehi et al., 2016). As for cell function, ADSCs possess excellent proliferation and differentiation ability, enabling a broad prospect in the treatment of peripheral nerve injury (Widgerow et al., 2013; Georgiou et al., 2015; Khalifian et al., 2015; Tomita et al., 2015; Salehi et al., 2016). In addition, ADSCs are capable of reducing inflammation around the injured nerve, not only promoting nerve regeneration but also inhibiting neural scarring (Balkin et al., 2014). Therefore, the marriage of nerve suture and simultaneous ADSCs injection into one modality is expected to be a novel potential for optimizing the therapy of peripheral nerve injury (Sullivan et al., 2016).

However, although stem cell therapy combined with nerve suture seems to be a feasible strategy offering shortened recovery time and improved neural function, the effectiveness and efficacy of which remain unknown. Moreover, little is known about the migration time, spatial distribution and cell retention of ADSCs during the nerve regeneration *in vivo*, which should play a crucial role in their ability to exert a therapeutic effect. Only when real-time visualization and monitoring of ADSCs after transplantation are realized, can the therapeutic regimen based on ADSCs-combined nerve suture be adjusted and optimized. In a word, an efficient imaging technology aiming for spatio-temporal analysis of ADSCs during nerve regeneration process can add new information for further studies in the treatment of peripheral nerve injury.

Recently, fluorescence imaging is becoming one of the most widely utilized techniques in the field of medical science, especially in molecular biology. The second near-infrared window (NIR-II, 1,000–1,700 nm) has made its debut by virtue of minimal scattering, high signal-to-noise ratio (SNR) and low self-fluorescence background compared with visible light and the first near-infrared window (NIR-I, 700–900 nm) (Welsher et al., 2011; Li et al., 2014; Hong et al., 2015; Chen et al., 2016; Wan et al., 2018). Lead sulfide quantum dots (PbS QDs) with emission in NIR-II window have been used to label Schwann cells when bound with motor neuron-specific protein Agrin to direct peripheral nerve imaging *in vivo* (Feng et al., 2019). Furthermore, stem cells were tracked by PbS QDs to observe the fate of injected cells during rotator cuff repair (Yang et al., 2019). Inspired by previous discoveries, the application of NIR-II imaging based on PbS QDs may hopefully serve as an excellent tool for monitoring ADSCs *in vivo* dynamically and in a real-time manner, generating a noteworthy potential for deciphering the underlying mechanism for peripheral nerve regeneration.

**Figure 1:**
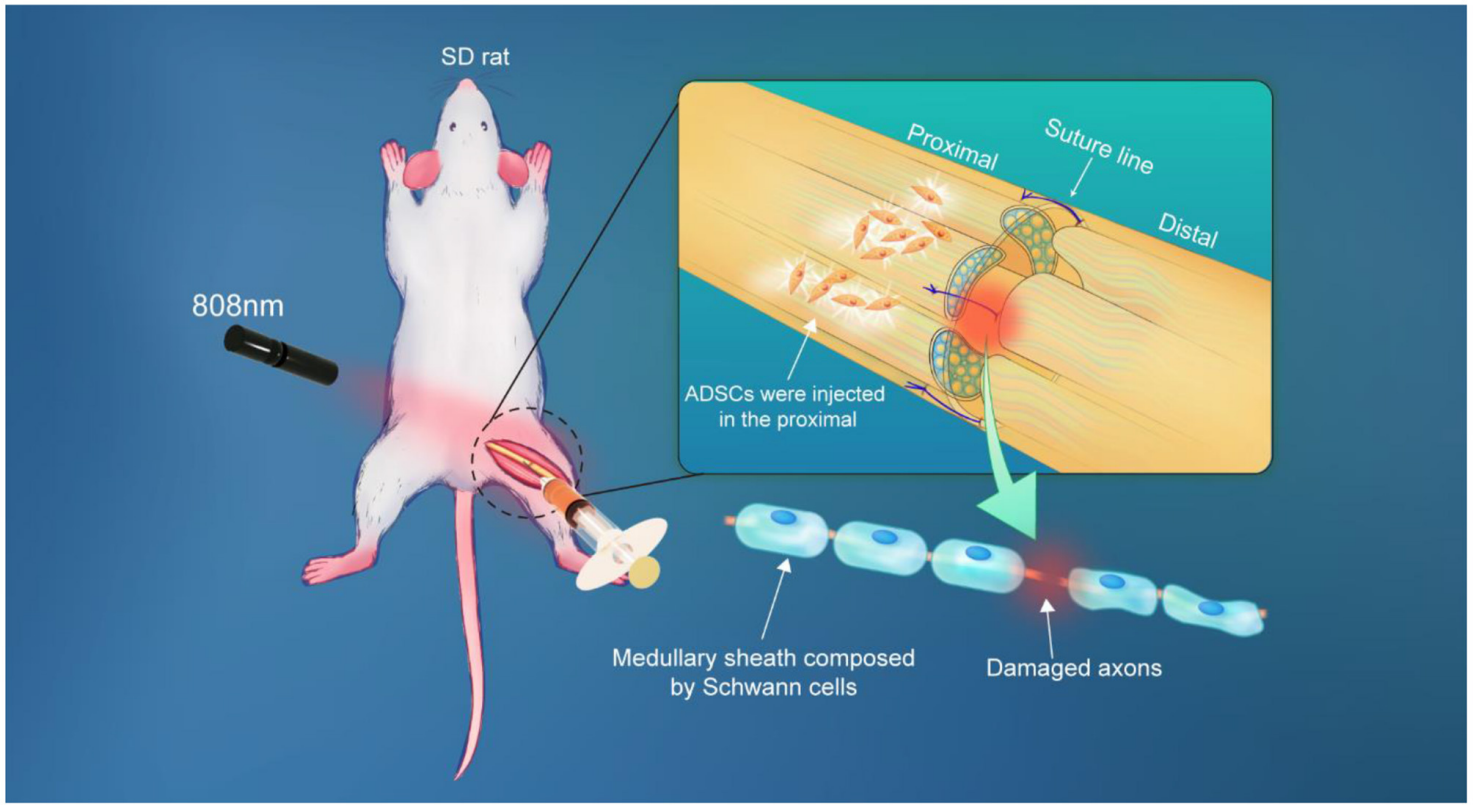
Schematic of the experimental process.

Here, longitudinal monitoring of injected ADSCs *in vivo* was performed in this work, providing a spatio-temporal map of ADSCs and allowing for in-depth interpretation of nerve regeneration after nerve suture combined with stem cell therapy (Figure 1). Firstly, our prepared PbS QDs were conjugated to Tat peptide, which acted as an effective vehicle for PbS QDs to label ADSCs as well as to track them *in vivo* (Kong et al., 2016). Then, fluorescence properties and stability of PbS QDs labeled ADSCs were studied both *in vitro* and *in vivo*. Subsequently, the PbS QDs labeled ADSCs were injected to rat models of sciatic nerve injury in combination with nerve suture, with the NIR-II fluorescence recorded in a time course *in vivo*. Furthermore, the efficacy of nerve suture combined with ADSCs injection was verified through electromyography and mechanical pain threshold. Additionally, pathological analysis was employed to testify the biosafety of PbS QDs labeled ADSCs. The current work proved nerve regeneration after the combined therapy and provided long-time and real-time details on the distribution, migration and clearance of the injected ADSCs, which contributed to an overall interpretation of the fate of injected cells and their therapeutic effect in combination with nerve suture in the treatment of peripheral nerve injury.

## Materials and Methods

### Reagents and Materials

All chemical reagents were utilized directly without further purification. Lead acetate trihydrate [Pb(OAc)_2_ ·3H_2_O, ≥99.9%], sodium sulfide non-ahydrate (Na_2_S·9H_2_O, ≥98.0%), Bovine pancreatic ribonuclease A (MW: 13.7 kDa, >70 U/mg), Fetal bovine serum (FBS) and NHS were purchased from Sigma-Aldrich. EDC and Sulfo-SMCC were purchased from Thermo Fisher Scientific. Tat peptide was customized synthesis in Ketai Biology. Sodium hydroxide (NaOH, 97%) was purchased from Maclin. Dulbecco's Modified Eagle Media: Nutrient Mixture F-12 (DMEM/F12) was obtained from Thermo Fisher Scientific. Cell Counting Kit-8 was purchased from Dojindo (Japan).

### Animals

All the experimental animals were 6 to 8 week old female Sprague-Dawley rats (weight: around 180 g) and were provided by the Animal Care Facility of Shanghai Jiao Tong University, School of Medicine (Shanghai, China). All animal experiments were carried out under aseptic conditions after the rats were anesthetized by intraperitoneal injection. The animal study protocol was approved by the Animal Care Committee of the Laboratory Animal from Fudan University (201903001S).

### Instrumentation

Deionized water was generated by an ELGA Purelab classic ultra violet filter system. The cultured cells were observed by Leica TCS SP8 microscope. The animal model establishment was completed under SMOIF SXP-1C microscope. The biosafety cabinet of 1300 SERIES A2 from Thermo SCIENTIFIC was used. Microwave synthesis was carried out in a microwave reactor (Discover, CEM, USA). The centrifugation was done using cence L530 centrifuge and AOSHENG mini-8k microcentrifuge. The NIR-II fluorescence emission spectrum was measured by NS1 NanoSpectralyzer fluorimetric analyzer with λex = 808 nm. The NIR-II fluorescence images were obtained from an *in vivo* imaging system (MARS, Artemis Intelligent Imaging, Shanghai, China). The excitation shadowless illumination was provided by a fiber-coupled 808 nm laser. Passing through NIR long-pass filter of 1,250 nm, emitted light was collimated by a 50 mm focal length SWIR lens (MARS-FAST, Artemis Intelligent Imaging, Shanghai, China). The fluorescence was then detected by a liquid-nitrogen-cooled InGaAs camera (NIRvana 640, Teledyne Princeton Instruments). Electromyography was tested by Powerlab from ADInstruments. Mechanical withdrawal threshold was measured by Electronic Von Frey from ugo basile.

### Synthesis of PbS QDs

The synthesis protocol of PbS QDs was based on the previously published paper (Kong et al., 2016). Firstly, 500 μL of completely dissolved Pb(CH3COO)_2_ (10 mM) and RNase A (50 mg/ml) solution were added into the reaction tube, respectively, and mixed. Then, 50 μL of NaOH (1 M) solution was used to adjust the pH of the system to 9–11. After that, 50 μL of Na_2_S (10 mM) was added. Before putting the reaction tube into the microwave reactor, a stir bar was added to help the mixture be heated evenly. The reaction parameters were set as dynamic mode, 30 W, 70°C, 30 s. The PbS QDs were stored at 4°C in dark after preparation.

### Cell Labeling by Tat-PbS QDs

The pH of PbS QDs solution was adjusted to 5-6 by NaOH and HCl. 0.24 mg of EDC and NHS were added into 1 mL of PbS QD solution and the mixture was vibrated at room temperature for 40 min. Next, PbS QDs were covalently bonded with Tat peptide using crosslinking reagent Sulfo-SMCC (Lei et al., 2008). After overnight reaction at 4°C, the mixture was centrifuged using ultrafiltration tube to remove the unbound free impurities. Then, the third generation of ADSCs were co-incubated with Tat-PbS QDs for about 3 h. After that, PBS (pH = 7.4) was used to wash away the Tat-PbS QDs that failed to label ADSCs.

### Cell Culture

ADSCs were isolated from inguinal adipose tissue of female Sprague-Dawley rats. The rats were anesthetized by intraperitoneal injection and then disinfected with 75% ethanol for 5–10 min. In an aseptic environment, the abdomenal skin of the rat was cut to expose the adipose tissues of the bilateral groin. Adipose tissues were clipped and small blood vessels were removed. Then the collected tissues were washed by PBS (pH = 7.4) for 3 times and cut into 1 mm × 1 mm × 1 mm pieces. The prepared I collagenase was used for digesting adipose tissue at 37°C for 1.5 h and the process was reversed every half an hour to make it completely digested. After that, the digested tissue was centrifuged (1,800 rpm, 8 min), the supernatant was discarded and the sediment was resuspended with PBS to original volume. After centrifugation again, the supernatant was discarded and the sediment was resuspended with DMEM/F12 medium again. Then the cells were filtered with a filter screen (70 μm) and transferred to a culture dish. The extracted cells were cultured in an incubator (37°C, 5% CO_2_). The culture medium was changed for the first time after 24 h and every 2–3 days afterwards.

### Preparation of Experimental Animal Models

In order to establish the rat model of sciatic nerve injury, the rats were placed properly in prone position after anesthesia. All instruments needed to be autoclaved in advance. The skin near the right lower limb was cut open to expose the sciatic nerve. The sciatic nerve was cut off from the middle of the sciatic nerve and immediately sutured. After that, the incision was closed and sterilized.

### *In vivo* Observation

The rats (*n* = 4)were anesthetized by intraperitoneal injection and fixed in prone position for NIR-II imaging. Ten microliter of the labeled ADSCs at different concentrations (1 × 10^5^, 5 × 10^4^, 5 × 10^3^, and 5 × 10^2^ cells) were first injected subcutaneously on the back for observation. The control group was treated with the same volume of PBS (pH = 7.4). Next, the PbS QDs labeled ADSCs were injected in sciatic nerve injury models. After the sutured sciatic nerve was exposed, 10 ul of the labeled ADSCs (5 × 10^5^ cells) were injected into the proximal side of the injured nerve. NIR-II fluorescence imaging system was then used to observe the migration of the injected cells with images and videos taken in a time course (0 d, 1 h, 1 d, 5 d, 7 d, 10 d, and 14 d post-injection). The PL intensity was measured from the site of the injury of collected NIR-II images using Image J.

### Electromyography

The compound muscle action potential (CMAP) of gastrocnemius was measured by electromyography. The rats (*n* = 4) were fixed in prone position after anesthesia and shaved near the lower limbs to expose the sciatic nerve after skin incision. CMAP was detected by biological signal acquisition and analysis system. The stimulation electrode was connected to the proximal end of the sciatic nerve, the receiving electrode was inserted into the gastrocnemius muscle, and the earth electrode was contacted with the tail of the rat. After electric stimulation, CMAP of gastrocnemius muscle was recorded and its amplitude was measured.

### Mechanical Pain Threshold Measurement

Electronic Von Frey test was used to measure mechanical withdrawal threshold of rats (*n* = 4). The touch stimulator transducer is mounted on a plexiglass rod to test the animal skin sensitivity. The center of the right limb of the rat was punctured for 15 s, and the rate of application of the force increased at a constant rate. The completion of each test may be indicated either by the sudden withdraw of the paw or lick the leg. Each rat was tested 3 times and the time interval is 20 s.

### Immunofluorescence Staining

The full-length sciatic nerves of rats were removed after anesthesia and then fixed in 4% paraformaldehyde. After that, they were embedded in paraffin and sectioned. Schwann cells were labeled with S-100 antibody, nerve fibers were labeled with neurofilament antibody and nuclei were stained with DAPI. Then, fluorescence were observed with images taken under fluorescence microscope.

### Statistical Analysis

All numerical data were presented as mean ± SD. Comparisons were performed using unpaired two-tailed Student's *t*-tests. *P* < 0.05 was considered statistically significant. The numerical data were analyzed by Graph Pad Prism 7.0.

## Results and Discussion

### Fluorescence Properties of PbS QDs + Tat and Labeling of Cells

**Figure 2:**
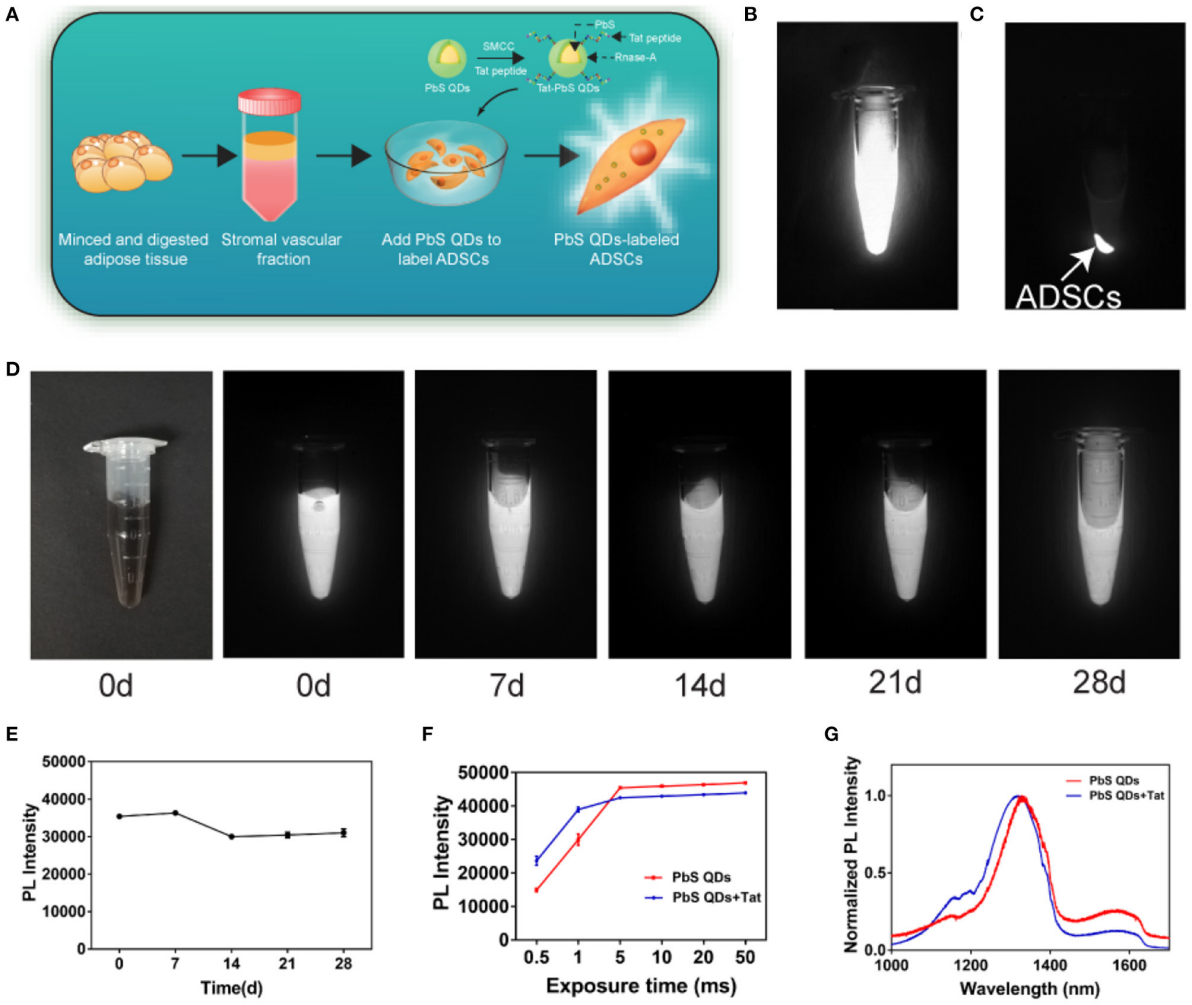
Fluorescence properties of PbS QDs+Tat and the labeling of ADSCs. **(A)** Schematic of synthesis of PbS QDs+Tat and labeling of ADSCs. **(B)** NIR-II imaging of PbS QDs labeled ADSCs in the state of suspension and **(C)** after centrifugation. **(D)** NIR-II imaging (λex: 808 nm, exposure time: 1 ms) of the same PbS QDs+Tat in a time course (0, 7, 14, 21, and 28 d post-preparation). **(E)** PL intensity of the same PbS QDs+Tat after storage. **(F)** PL intensity of PbS QDs and PbS QDs+Tat under different exposure time. **(G)** Normalized PL spectra of PbS QDs and PbS QDs +Tat.

The PbS QDs were first prepared according to the previous protocol (Kong et al., 2016). Then, the as-prepared PbS QDs were conjugated to Tat peptide using SMCC-EDC method (Chen et al., 2014). The schematic of synthesis of PbS QDs+Tat and labeling of ADSCs was shown in Figure 2A. Fluorescence properties of pure PbS QDs were verified in Supplementary Figure 1. As shown in Supplementary Figure 2, NIR-II fluorescence images of 1 ml of PbS QDs+Tat were obtained with an excitation wavelength of 808 nm under increasing exposure time (0.5, 1, 5, 10, 20, and 50 ms). The liquid could be clearly observed even at a short exposure time of 0.5 ms, confirming excellent fluorescence intensity and sensitivity as reported in the previous study (Yang et al., 2019). Moreover, it was shown that the PL intensity of PbS QDs+Tat increased with exposure time from 0.5 to 5 ms and remained high from 5 to 50 ms, and was close to the PL intensity of PbS QDs (Figure 2F). Therefore, it was envisaged that exposure time over 5 ms would be mighty enough for *in vitro* observation.

To investigate the stability of the freshly prepared PbS QDs+Tat, the same tube of PbS QDs+Tat was stored at 4°C in darkness and was photographed with NIR-II imaging system at 0, 7, 14, 21, and 28 d post-preparation (Figure 2D). The PL intensity remained nearly the same as freshly prepared without drastic fluctuation after 28 days (Figure 2E), verifying robust photostability of PbS QDs+Tat as reported (Yang et al., 2019). Furthermore, the normalized PL spectra of PbS QDs+Tat and PbS QDs were measured and compared in Figure 2G. A minor blueshift of the emission peak in the spectra was observed after conjugation, which could result from a larger spacing of the complex structure after combining with a peptide (Kong et al., 2010; Pan et al., 2015; Zhao et al., 2019). Nevertheless, both the curves displayed an emission peak (around 1,300 nm) locating within the range of NIR-II window, indicating that PbS QDs+Tat had comparable fluorescence properties with PbS QDs.

The PbS QDs+Tat of robust fluorescence properties and stability were then applied to label ADSCs (30 ug/mL, isolated from SD rats). The extraction process of ADSCs was shown in Supplementary Figure 3 and the cytotoxicity of PbS QDs was assessed (Supplementary Figure 4). After incubation overnight, sufficient labeling of the cells was achieved. The NIR-II fluorescence signals detected in the cell sediments showed evidence for successful labeling of ADSCs by PbS QDs (Figures 2B,C). In summary, NIR-II fluorescence imaging based on PbS QDs demonstrated favorable fluorescence properties and were capable of labeling ADSCs *in vitro* after conjugation with Tat. The results suggested that PbS QDs had promising potential for tracking ADSCs *in vivo*.

### *In vivo* Sensitivity and Stability of ADSCs Labeled With PbS QDs

**Figure 3:**
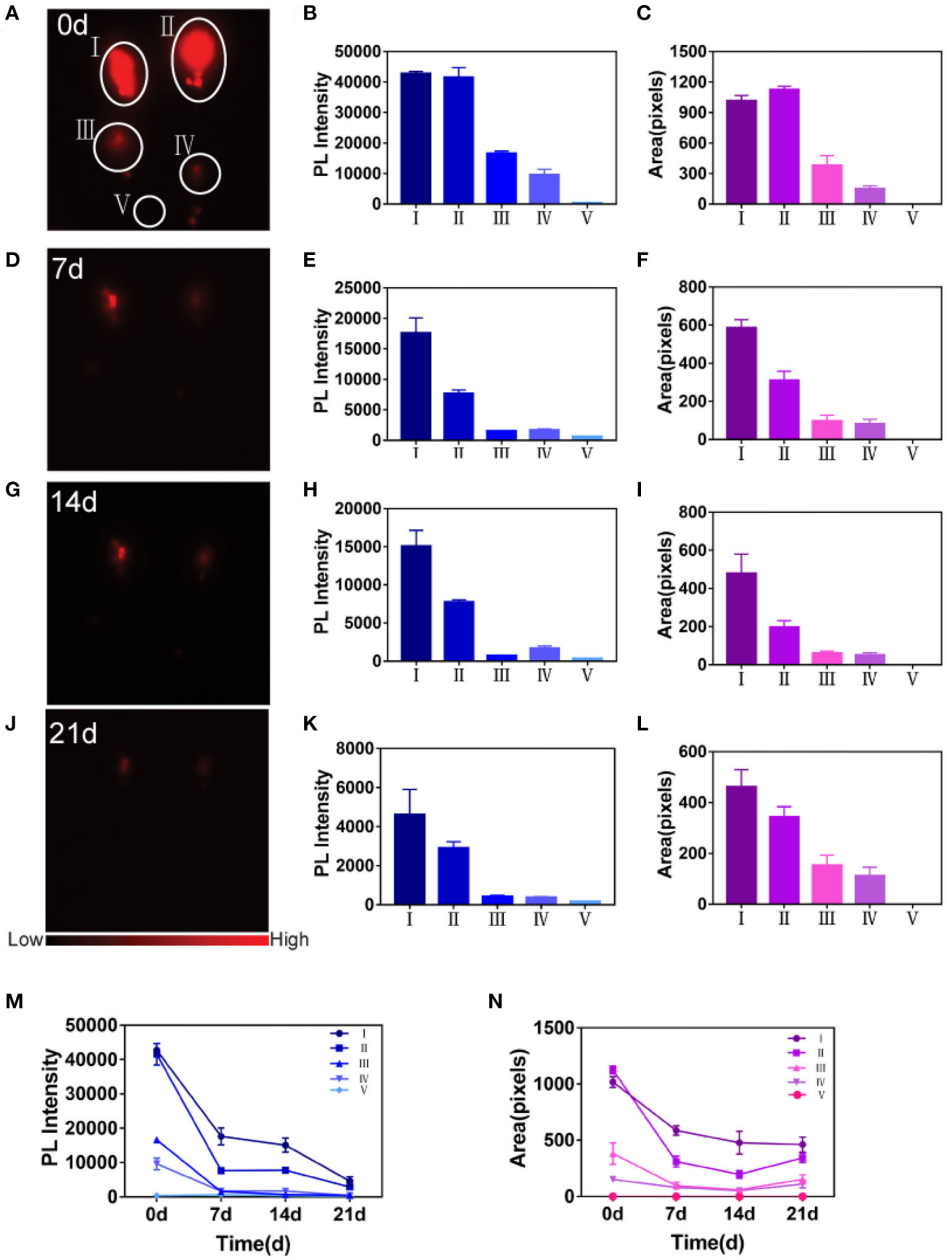
Detection of sensitivity and stability of PbS QDs labeled ADSCs *in vivo*. **(A,D,G,J)** NIR-II imaging of PbS QDs labeled ADSCs (1 × 10^5^, 5 × 10^4^, 5 × 10^3^, and 5 × 10^2^ cells) and PBS *in vivo* in a time course (0, 7, 14, and 21 d post-injection). **(B,E,H,K)** PL intensity measured from **(A,D,G,J)**. **(C,F,I,L)** Fluorescence area measured from **(A,D,G,J)**. **(M)** Longitudinal comparison of PL intensity measured from **(A,D,G,J)**. **(N)** Longitudinal comparison of fluorescence area measured from **(A,D,G,J)**.

Despite the effectiveness of labeling ADSCs by PbS QDs *in vitro*, further validation of sensitivity and stability of labeled ADSCs *in vivo* was required. As shown in Figures 3A,D,G,J, four concentrations of PbS QDs labeled ADSCs (1 × 10^5^, 5 × 10^4^, 5 × 10^3^ and 5 × 10^2^ cells) and PBS as control were subcutaneously injected into five different points on the dorsum of a rat with NIR-II images recorded in a time course (0, 7, 14, and 21 d post-injection). The corresponding PL intensity and fluorescence area were measured in Figures 3B,C,E,F,H,I,K,L.

Overall, the fluorescence signals decreased gradually at the four points with injected PbS QDs labeled ADSCs as observation time increased (Figures 3A,D,G,J). The fluorescence of the injected PBS (the control group) was almost the same as the background. At 0 d post-injection, the PL intensity exhibited a linear behavior with cell concentration (Figure 3B), and fluorescence signals could only be detected at the first two points with 1 × 10^5^ cells and 5 × 10^4^ cells at 21 d post-injection (Figure 3J). These results confirmed again that the ADSCs were successfully labeled and could be tracked *in vivo*.

Specifically, the point with the highest cell concentration (1 × 10^5^) showed the highest fluorescence signals for the longest time, demonstrating that ADSCs could be best detected at this concentration using the NIR-II imaging system (Figures 3A,D,G,J). Furthermore, the longitudinal changes of PL intensity and fluorescence area were shown in Figures 3M,N across the five concentrations of PbS QDs labeled ADSCs. Both PL intensity and fluorescence area showed that ADSCs at a concentration of 1 × 10^5^ cells decreased slowly, suggesting the longest retention at the injected site with durable fluorescence throughout the 21 day observation. Moreover, it was reported that concentration around 10^5^-10^6^ ADSCs were suitable for nerve regeneration (Tremp et al., 2018; Rodríguez Sánchez et al., 2019). Therefore, 1 × 10^5^ cells was considered the optimal concentration and was chosen to complete the subsequent experiment *in vivo*.

Thus, desirable sensitivity and stability of PbS QDs-based NIR-II imaging both *in vitro* and *in vivo* proved that it was promising for visualization and monitoring of the long-time translocation of ADSCs for medical applications *in vivo*.

### Real-Time and Dynamic NIR-II Fluorescence Imaging of ADSCs *in vivo*

**Figure 4:**
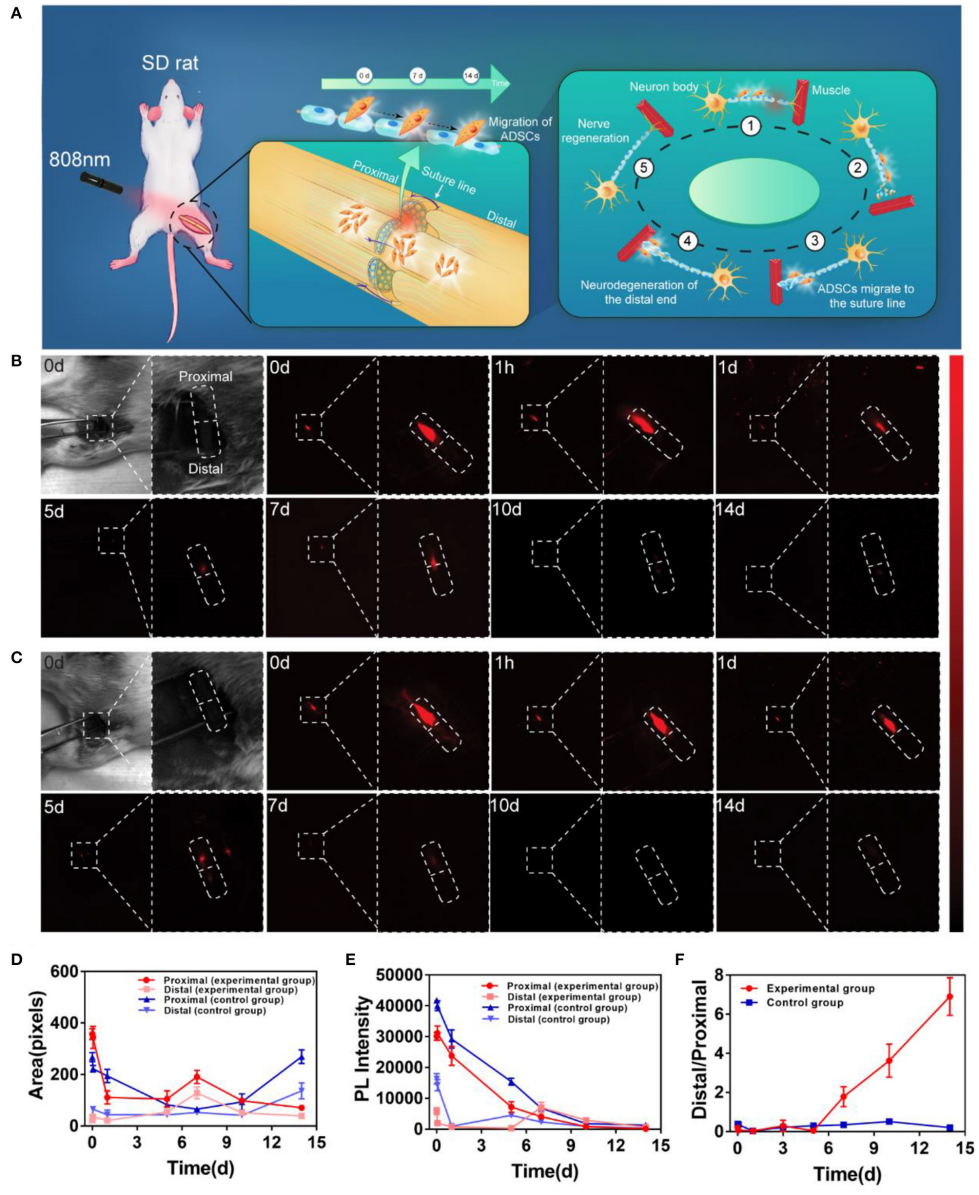
*In vivo* distribution and migration of PbS QDs labeled ADSCs in a rat model of sciatic nerve injury. **(A)** Schematic illustration of the *in vivo* NIR-II imaging strategy. **(B)** Bright field photograph of the region of interest and NIR-II imaging of the experimental group (injected with PbS QDs labeled ADSCs) in a time course (0 d, 1 h, 1 d, 5 d, 7 d, 10 d, and 14 d post-injection). **(C)** Bright field photograph of the region of interest and NIR-II imaging of the control group (injected with pure PbS QDs) in a time course (0 d, 1 h, 1 d, 5 d, 7 d, 10 d, and 14 d post-injection). **(D)** Fluorescence area and **(E)** PL intensity of the proximal and distal sections of the sciatic nerve. **(F)** The ratio of PL intensity in distal/proximal nerve sections in the experimental group and the control group.

The schematic of *in vivo* observation was shown in Figure 4A. During the observation, ADSCs were first labeled with PbS QDs *in vitro*. After the sciatic nerve of the rat was exposed, cut open and sutured in the middle (Supplementary Figure 5), 10 ul of the PbS QDs labeled ADSCs (5 × 10^5^ cells in concentration) were injected into the proximal section of the injured nerve. Then, longitudinal observation of the same area of interest by NIR-II imaging was initiated, with images and movies (Supplementary Movie 1) obtained in a time course (0 d, 1 h, 1 d, 5 d, 7 d, 10 d, and 14 d post-injection).

Strong NIR-II fluorescence signals were clearly observed at the proximal section of the sutured sciatic nerve at 0 d post-injection in both experimental and control group (Figures 4B,C). This indicated that most of the PbS QDs labeled ADSCs were instantly confined in the proximal injection site of the sutured nerve at first. Then, in the experimental group, the fluorescence signals remained detectable and moved gradually from the proximal section to the distal section of the nerve throught the 14-day observation, suggesting migration of ADSCs from the proximal section (0 d post-injection) of the nerve to the sutured site (7 d post-injection), and then to the distal section (14 d post-injection) (Figure 4B). In contrast, the fluorescence signals in the control group decreased steadily around the injection site without obvious change of location (Figure 4C). It is worth noting that the nerve healing process leads to release of numerous chemokines to promote nerve regeneration (Terenghi, 1999). Therefore, it was postulated that chemotactic factors released during nerve regeneration might guide the migration of ADSCs to the distal section of the injured nerve, where the Wallerian degeneration began and the regeneration from the proximal section would gradually spread to Fawcett and Keynes (1990) and Chen et al. (2007).

To quantitatively evaluate the migration of injected ADSCs, PL intensity and fluorescence area were measured in Figures 4D,E. Compared with the control group, the PL intensity of the proximal section of the nerve decreased steadily accompanied by gradual increase in the distal section in experimental group (Figure 4D). As for fluorescence area, although a decrease in the proximal section of the nerve was observed in both groups, the reason behind could be different. For the experimental group, the fluorescence signals appeared in the distal section, which indicated the migration of ADSCs. However, no fluorescence signal was detected in the distal section of the control group, suggesting the metabolism of pure PbS QDs. To demonstrate the result more clearly, the ratio of distal/proximal PL intensity of both groups was calculated. As shown in Figure 4F, the increasing ratio revealed an accumulation of PbS QDs labeled ADSCs in the distal section of the nerve accompanied by reduction of ADSCs in the proximal section (where the cells were initially injected) of the experimental group in comparison with the control group. These results were believed to have shown that a tempo-spatial distribution of ADSCs in rats with sciatic nerve injury was visualized by PbS QDs based NIR-II imaging *in vivo*. Therefore, it was reasonable to regard PbS QDs as a candidate for monitoring the migration of ADSCs in nerve regeneration.

### Functional Recovery After Combined Treatment

**Figure 5:**
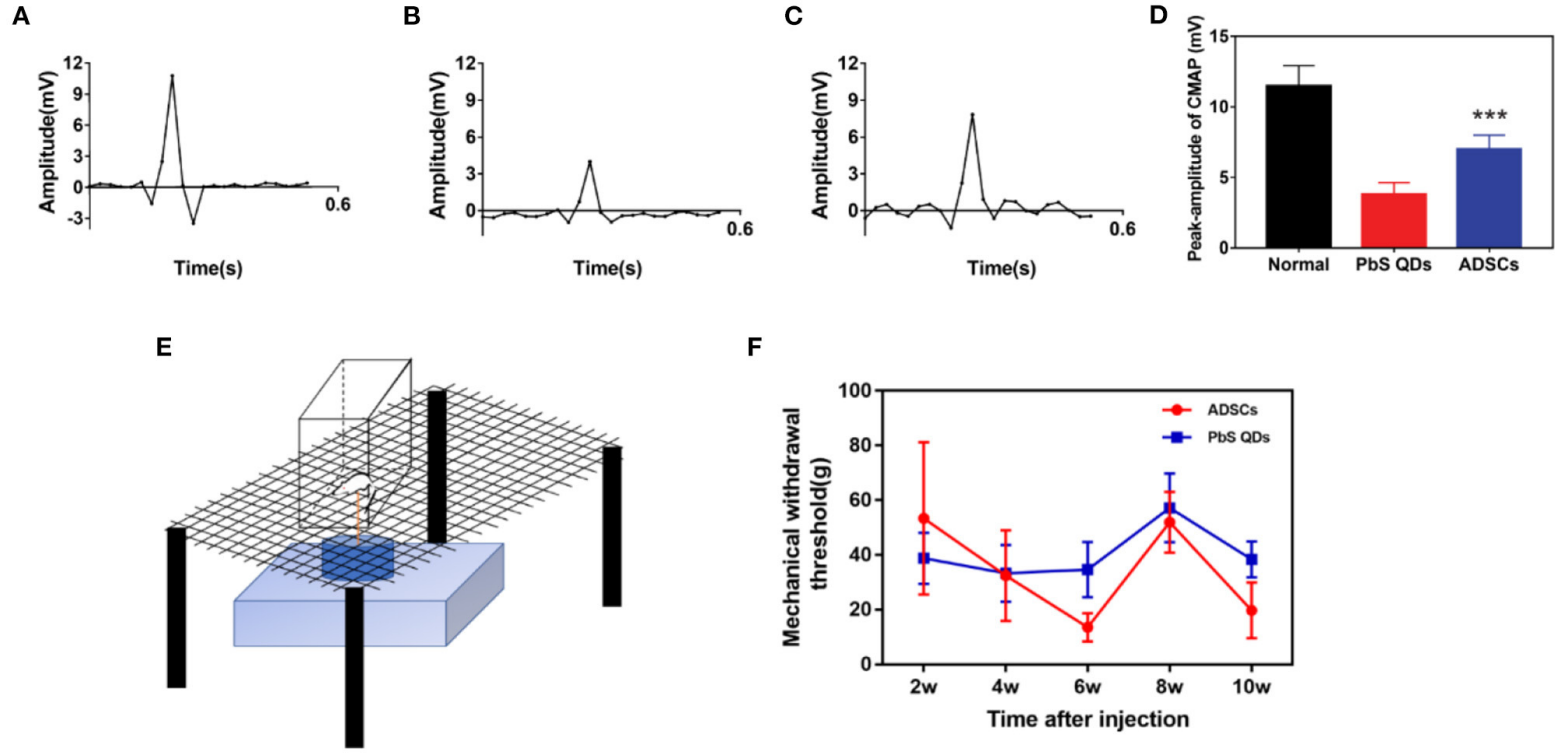
Functional examination through electromyography and mechanical pain threshold. Electromyography of **(A)** healthy rats, **(B)** rats with PbS QDs injection, and **(C)** rats with PbS QDs labeled ADSCs injection at 3 m post-injection. **(D)** Histogram of signal intensity measured from **(A–C)**. **(E)** Pattern diagram of Electronic Von Frey in rat. **(F)** Measurements of the mechanical withdrawal threshold of rat taken after injury (***P < 0.001, vs. PbS QDs).

The therapeutic effect of PbS QDs labeled ADSCs were verified by electromyography and mechanical pain threshold (Figure 5). Confirmed by electromyography, the rats treated with PbS QDs labeled ADSCs (Figure 5C) demonstrated stronger electrical signals than those injected with pure PbS QDs (Figure 5B), illustrating a superior recovery of neural function after ADSCs treatement. Although the signals were still weaker than that of healthy control (Figure 5A), this might be due to an insufficiency of recovery time. In addition, quantitative analysis was shown in Figure 5D, indicating better recovery of the combined therapy. Besides, the mechanical pain threshold was measured to prove the functional improvement in the experimental group (Figure 5E). As shown in Figure 5F, the mechanical withdrawal threshold of the rats with combined therapy was nearly the same as the control group at 4 w post-injection and kept at a low level afterwards. This result suggected that these rats regained functional recovery with superior pain sensation at an earlier time post-operative, showing the therapeutic effect of injected ADSCs. Furthermore, pathological analysis with immunofluorescence staining of the injured nerve from normal rats and rats after surgery was performed, indicating pure surgery was unable to achieve an optimal histological recovery (Supplementary Figure 5). Thus, nerve suture with ADSCs injection was proved to result in satisfactory functional recovery and nerve regeneration, and at the same time verified the migration of ADSCs observed with NIR-II imaging system.

### Biosafety of PbS QDs

**Figure 6:**
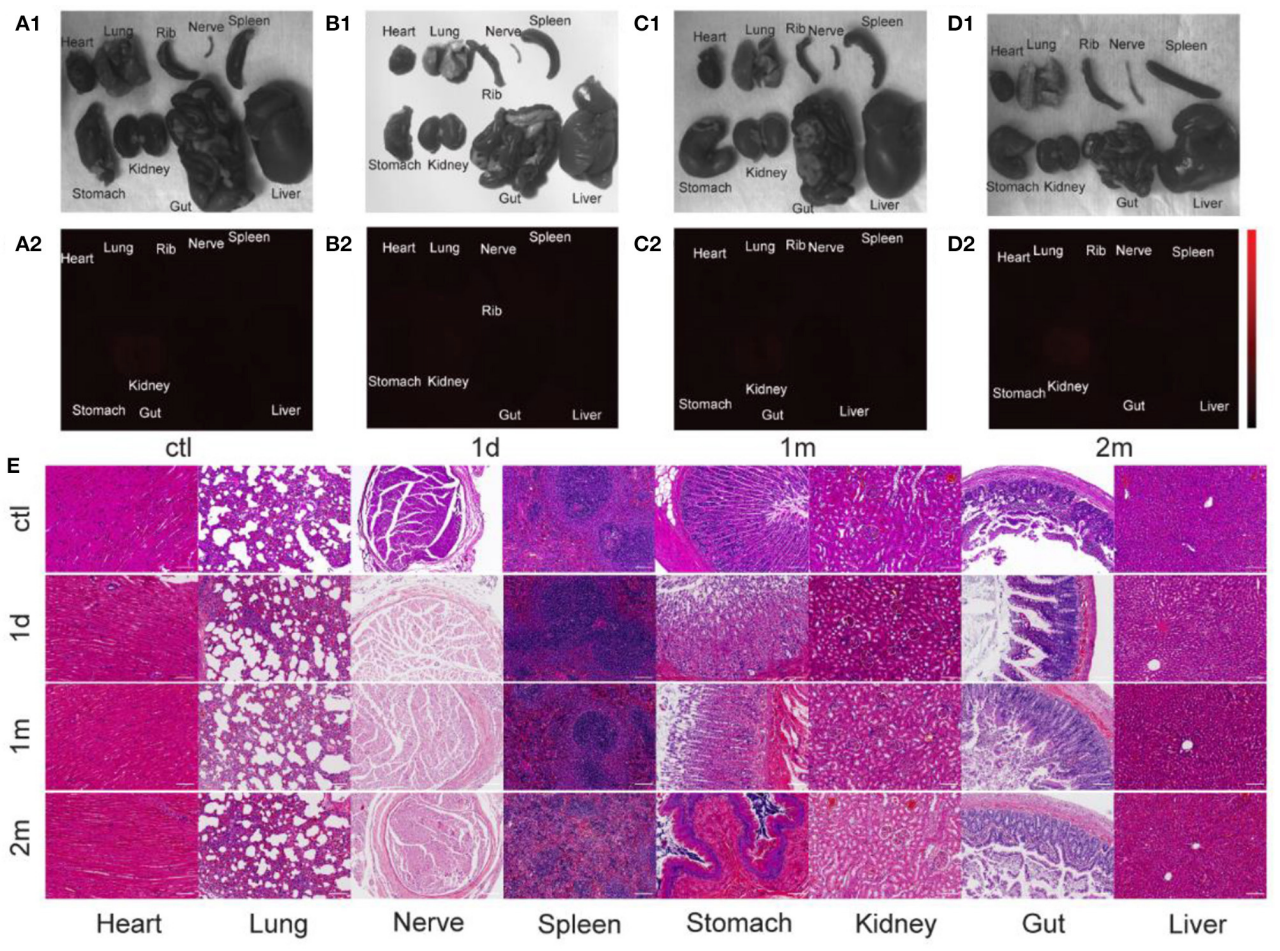
Biosafety of PbS QDs labeled ADSCs. **(A1–D1)** Bright field photographs and **(A2–D2)** NIR-II fluorescence images of major organs harvested from the rats in the control group and experimental group at 1 d, 1 m, and 2 m post-injection. **(E)** Representative HandE staining of major organs (collected and at 1 d, 1 m, and 2 m post-injection) of the rats injected with PbS QDs labeled ADSCs and the control group (scale bars represent 100 μm).

Major organs of the rats in the experimental group were harvested at at 1 d, 1 m, 2 m post-injection to study the biosafety of PbS QDs labeled ADSCs. The bright field photographs of freshly dissected organs were shown in Figures 6A1–D1, and no obvious abnormality was discovered compared with the control group. As shown in Figures 6A2–D2, most of the organs, including heart, lung, nerve, spleen, stomach, kidney, gut and liver showed subtle NIR-II signals, indicating the clearance of injected cells out of the body. Furthermore, the biosafety was further testified by hematoxylin and eosin (H&E) staining (Figure 6E). The representative images demonstrated major organs collected at 1 d, 1 m, and 2 m post-injection from the rats injected with PbS QDs labeled ADSCs. No noticeable injury or inflammation was observed, which further ensured the biosafety of PbS QDs labeled ADSCs.

## Conclusion

In this study, *in vivo* dynamic monitoring of ADSCs based on NIR-II fluorescence imaging combined with nerve suture during sciatic nerve regeneration was successfully achieved. By labeling ADSCs with PbS QDs, accurate and reliable records of the migration and distribution of ADSCs in nerve regeneration were obtained. The *in vivo* results demonstrated that the fluorescence signals of ADSCs in the proximal section of the sutured nerve decreased steadily accompanied by gradual increase in the distal section during a 14 day observation, indicating migration of ADSCs from the proximal section (0 day post-injection) of the nerve to the sutured site (7 days post-injection), and then to the distal section (14 days post-injection). The electromyography and mechanical pain threshold showed nerve recovery functionally. Our findings indicated that nerve suture combined with ADSCs injection resulted in nerve regeneration and neurofunctional recovery, the process of which could be precisely monitored by NIR-II fluorescence imaging *in vivo*, displaying considerable potential for future peripheral nerve injury treatment under real-time monitoring *in vivo*.

## Data Availability Statement

The original contributions presented in the study are included in the article/Supplementary Material, further inquiries can be directed to the corresponding author/s.

## Ethics Statement

The animal study was reviewed and approved by Ethics Committee of FudanUniversity.

## Author Contributions

SD and SF performed the experiment and wrote the article. YC, MC, YY, JZ, and HL helped perform the experiment. XL, LJ, and XY assisted in the article polishment. YH, JC, and YW were in charge of the whole study. All authors contributed to the article and approved the submitted version.

## Conflict of Interest

The authors declare that the research was conducted in the absence of any commercial or financial relationships that could be construed as a potential conflict of interest.
