# Supplementary material for: Nerve Suture Combined With ADSCs Injection Under Real-Time and Dynamic NIR-II Fluorescence Imaging in Peripheral Nerve Regeneration in vivo
Source: Front Chem. 2021 Jul 14;9:676928. doi: 10.3389/fchem.2021.676928 (PMC8317167; doi:10.3389/fchem.2021.676928)
Supplement: Supplementary file 2 [file Data_Sheet_1.docx]

**Nerve Suture Combined with ADSCs Injection under Real-time and Dynamic NIR-II Fluorescence Imaging in Peripheral Nerve Regeneration *in vivo***

*Shixian Dong^†^, Sijia Feng^†^, Yuzhou Chen, Mo Chen, Yimeng Yang, Jian Zhang, Huizhu Li, Xiaotong Li, Liang Ji, Xing Yang, Yuefeng Hao^*^, Jun Chen^*^, Yan Wo^*^*

**Supporting Information**

**
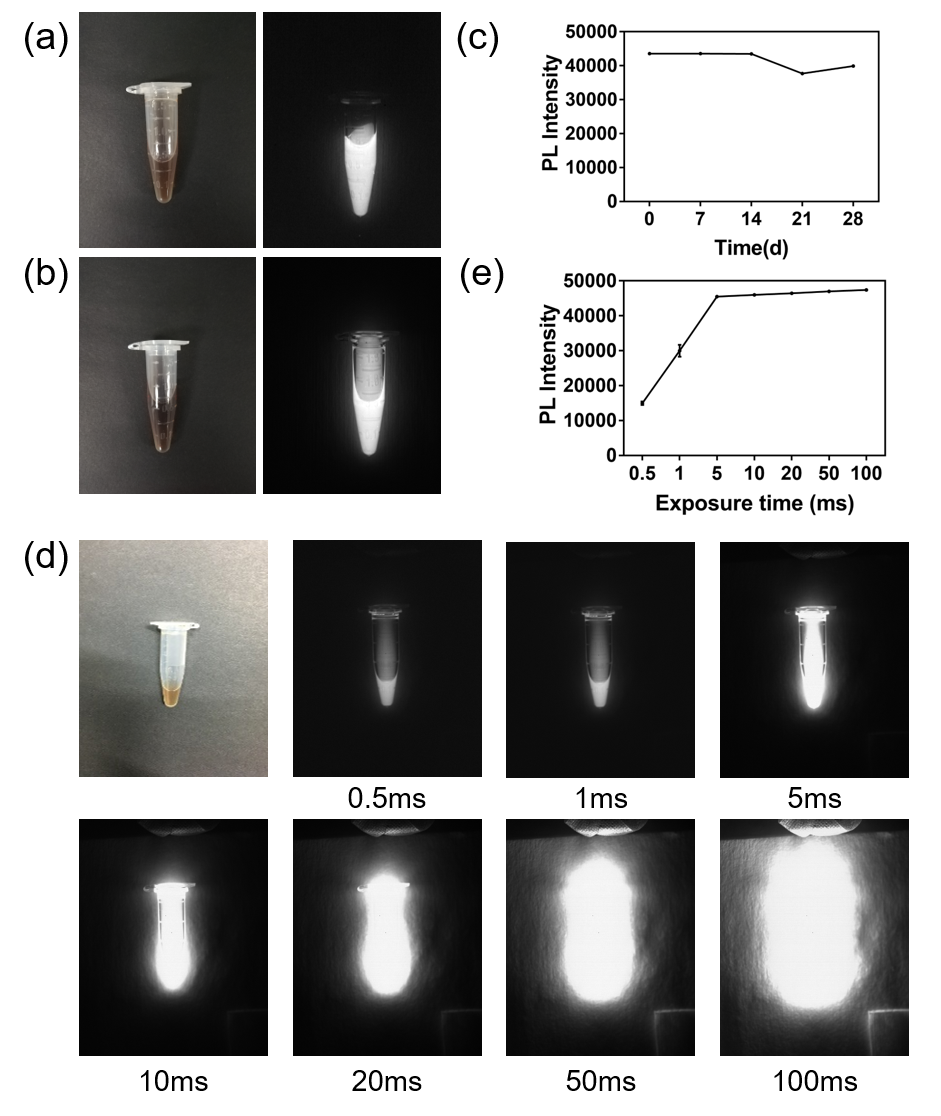
**

**Figure S1. Fluorescence properties of PbS QDs and PbS QDs+Tat.** White light photos and NIR-II images (λex: 808nm, exposure time: 1ms) of a) PbS QDs and b) PbS QDs +Tat. c) PL intensity of the same PbS QDs after storage for 4 weeks. d) White light photo and NIR-II images of PbS QDs under different exposure time (0.5ms, 1ms, 5ms, 10ms, 20ms, 50ms and 100ms). e) PL intensity measured from d).

**
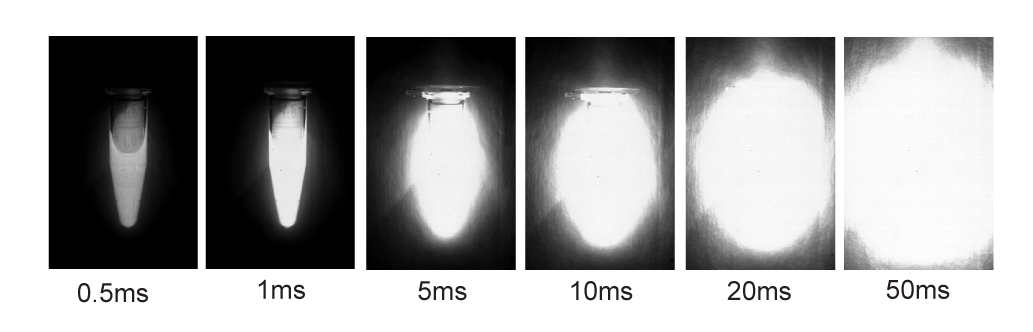
**

**Figure S2.** NIR-II imaging (λex: 808nm, exposure time: 0.5ms, 1ms, 5ms, 10ms, 20ms and 50ms) of PbS QDs+Tat.


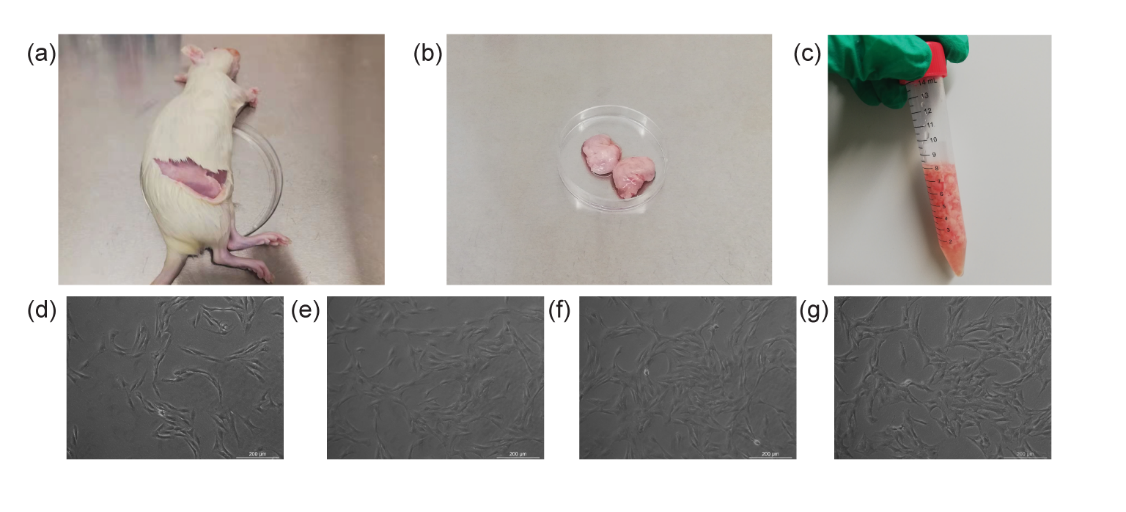


**Figure S3. Extraction and culture of rat ADSCs.** a) Exposure of adipose tissue in groin of rats. b) Isolated adipose tissue. c) Digestion of adipose tissue. d-g) Morphology of primary ADSCs and p1-p3 ADSCs in culture under a microscope.


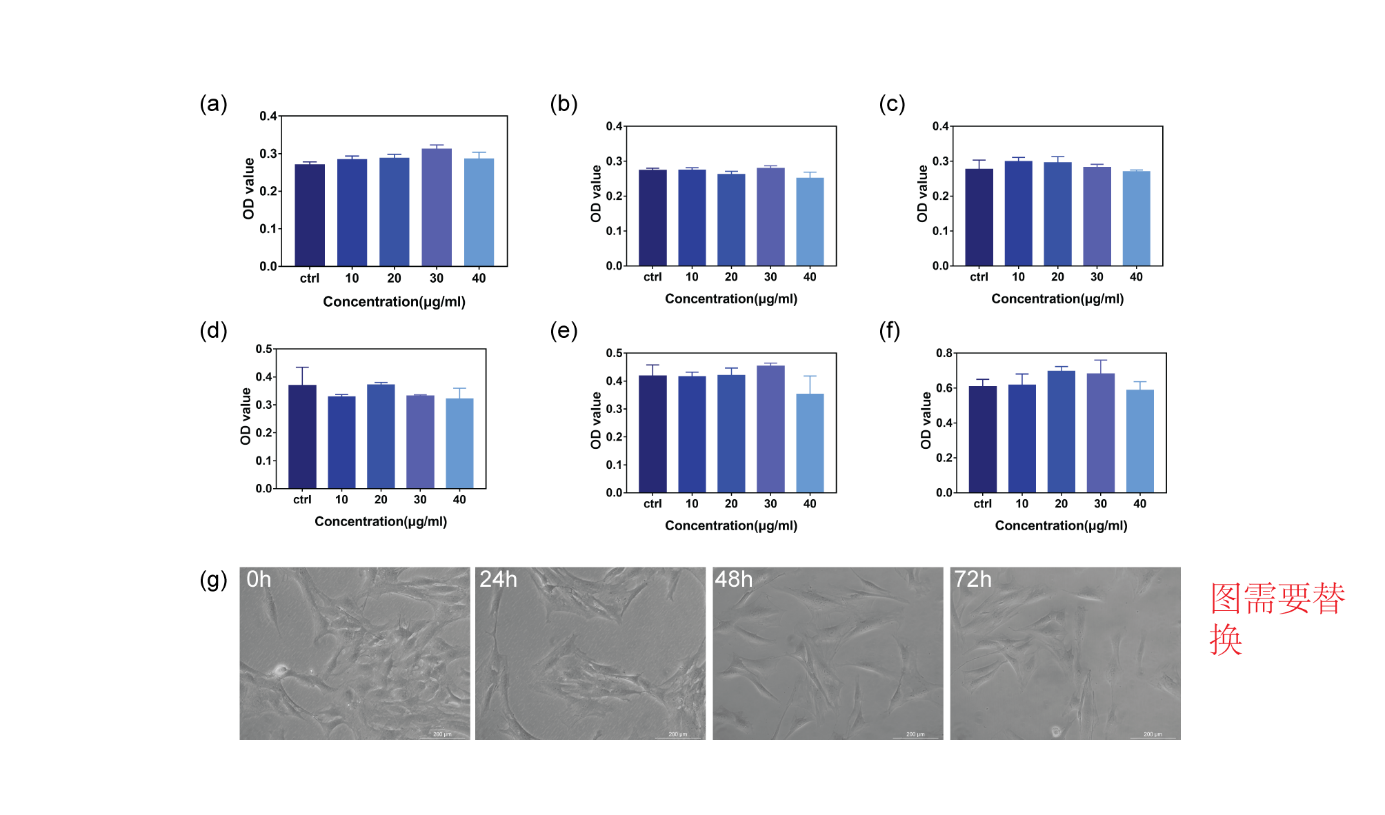


**Figure S4. Cytotoxicity of PbS QDs assessed with Cell Counting Kit.** ADSCs treated with different concentrations (10, 20, 30 and 40μg/mL) of PbS QDs. a-f) Cell viability and g) cell morphology (30μg/mL) observed in a time course (1h, 6h, 12h, 24h, 48h and 72h post-culture).


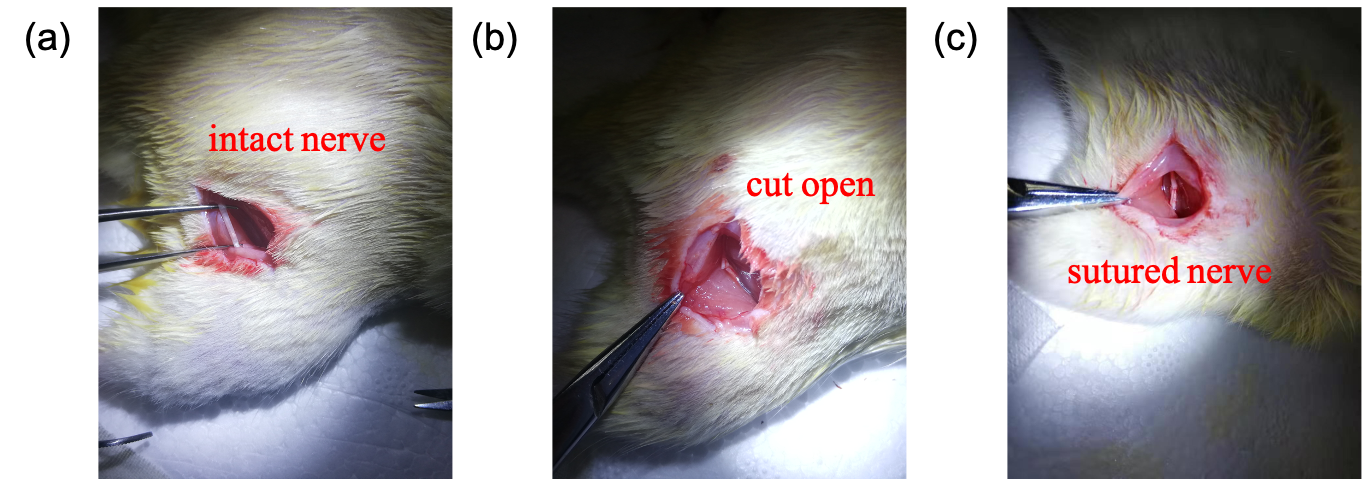


Figure S5. White light photo of the surgery process. a) Exposure of the sciatic nerve. b) Cut open of the sciatic nerve. c) Suture of the sciatic nerve.


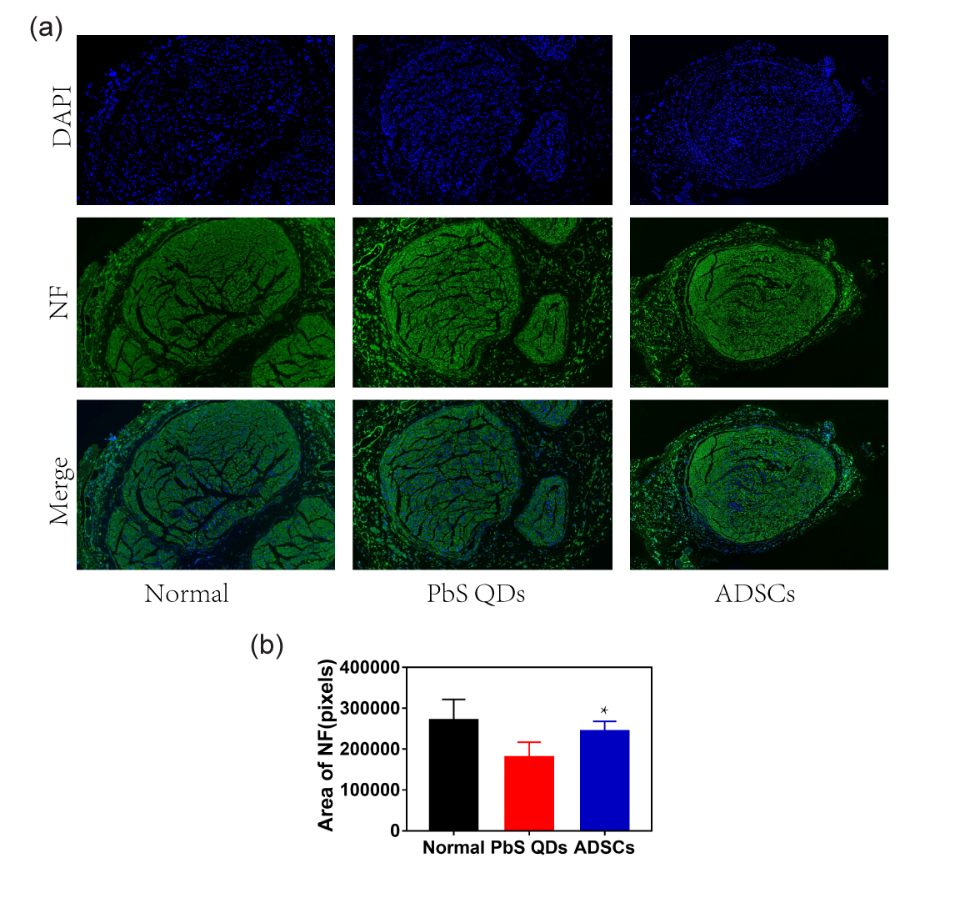


**Figure S6.** Immunofluorescence staining of sciatic nerve tissue sections of normal rats and rats injected with PbS QDs or PbS QDs labeled ADSCs at 3m post-injury. a) Representative immunofluorescence staining images with neurofilaments (NF, green) stained (scale bars represent 100μm). Fluorescence quantification of b) neurofilaments measured from a). (Asterisk (*) indicates P < 0.05, vs PbS QDs.)


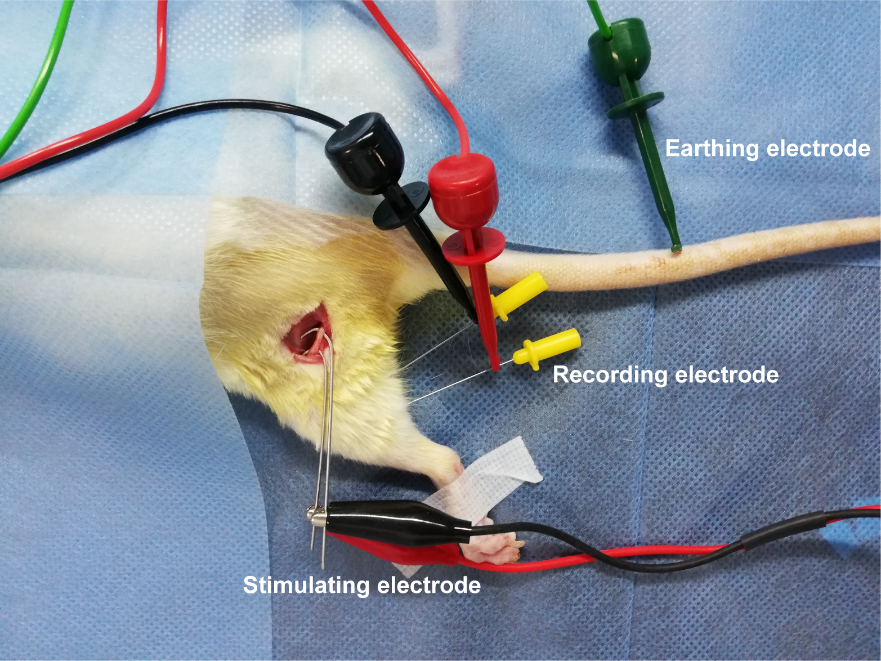


**Figure S7.** Photo of the rat during electromyography.
